# Gel/hydrogel‐based in situ biomaterial platforms for cancer postoperative treatment and recovery

**DOI:** 10.1002/EXP.20220173

**Published:** 2023-05-31

**Authors:** Yuzhao Feng, Zhan Zhang, Wei Tang, Yunlu Dai

**Affiliations:** ^1^ Cancer Centre and Institute of Translational Medicine Faculty of Health Sciences University of Macau Macau SAR China; ^2^ MoE Frontiers Science Center for Precision Oncology University of Macau Macau SAR China; ^3^ Departments of Pharmacy and Diagnostic Radiology Nanomedicine Translational Research Program Faculty of Science and Yong Loo Lin School of Medicine National University of Singapore Singapore

**Keywords:** gel, hydrogel, metastasis, recovery, recurrence, tumor postoperative treatment

## Abstract

Tumor surgical resection is the major strategy for cancer treatment. Meanwhile, perioperative treatment especially the postoperative adjuvant anticancer strategies play essential roles in satisfying therapeutic results and rapid recovery. Postoperative tumor recurrence, metastasis, bleeding, inter‐tissue adhesion, infection, and delayed wound healing are vital risks that could lead to poor prognosis or even treatment failure. Therefore, methods targeting these postoperative complications are in desperate need. In situ biomaterial‐based drug delivery platforms are promising candidates for postoperative treatment and recovery, resulting from their excellent properties including good biocompatibility, adaptive shape, limited systemic effect, designable function, and easy drug loading. In this review, we focus on introducing the gel/hydrogel‐based in situ biomaterial platforms involving their properties, advantages, and synthesis procedures. Based on the loaded contents in the gel/hydrogel such as anticancer drugs, immunologic agents, cell components, and multifunctional nanoparticles, we further discuss the applications of the in situ platforms for postoperative tumor recurrence and metastasis inhibition. Finally, other functions aiming at fast postoperative recovery were introduced, including hemostasis, antibacterial infection, adhesion prevention, tissue repair, and wound healing. In conclusion, gel/hydrogel is a developing and promising platform for postoperative treatment, exhibiting gratifying therapeutic effects and inconspicuous toxicity to normal tissues, which deserves further research and exploration.

## Graphical abstract

Gel/Hydrogel‐based in situ biomaterial platforms are promising candidates for cancer postoperative treatment and recovery, which decrease the risk of poor prognosis caused by postoperative complications. This review summarized the properties and synthesis procedures of gel/hydrogel‐based biomaterials and highlighted their advantages in postoperative tumor recurrence and metastasis inhibition by various drug loading and enhanced local delivery. Other multifunctional applications of gel/hydrogel for disease recovery were also introduced, including postoperative hemostasis, antibacterial infection, adhesion prevention, tissue repair, and wound healing.

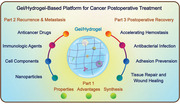

## 1 INTRODUCTION

Despite the constant improvement of modern medicine, cancer is difficult to cure completely, resulting in one of the leading causes of death worldwide.^[^ ^1^ ^]^ Surgical resection of tumor is currently the major treatment method for cancer patients. However, distressing postoperative tumor recurrence and metastasis severely affect patients’ quality of life and decrease long‐term survival rate. One major challenge is that there remain residual tumor cells after surgical resection, which may hide deep in the surrounding tissues or be left in the margin tissues at the excised tumor site because of their indistinguishable size. Following the bleeding blood during operation, they may proliferate and lead to seeding metastasis in the adjacent tissues, or get access into the circulation and metastasize to distant sites, resulting in tumorigenesis and recurrence.^[^ ^2^ ^]^ Besides, researchers have found that surgery itself may accelerate tumor growth by inducing catecholamine surge that results in β_2_‐adrenergic signaling culmination and the production of growth factors and proangiogenic factors (e.g., vascular endothelial growth factor, VEGF) for wound healing.^[^ ^3^ ^]^ Sustained elevation of these factors can initiate the proliferation and transformation of occult non‐angiogenic tumor cells into angiogenic tumors, which are well controlled in dormant stage in healthy bodies.^[^ ^3^ ^]^ For patients suffering from operative tumors, to decrease the chance of tumor postoperative recurrence and metastasis, some general follow‐up adjuvant therapies are suggested to be conducted after tumor resection to remove residual tumor cells, such as chemotherapy, immunotherapy, and targeted therapy.^[^ ^4^, ^5^ ^]^ However, these traditional therapies are mainly administrated systemically, which not only causes non‐selective damage to all cells, leading to severe side effects to normal tissues, but also show short half‐life blood circulation and low drug bioavailability, which could further limit the therapeutic effect.^[^ ^6^ ^]^ Therefore, developing new strategies for tumor postoperative treatment brooks no delay.

A biomaterial refers to an engineered non‐viable substance used in medical devices or living organisms that can interact with the biological system and achieve some expected functions.^[^ ^7^, ^8^ ^]^ From metal to ceramic, from polymer to composite, from synthetic to natural, from inert to bioactive,^[^ ^9^ ^]^ various properties of different materials give rise to multiple functions of biomaterials, which establishes its irreplaceable roles in modern biomaterial‐based medicine. With the increasing degree of sophistication, biomaterials (e.g., content lenses, artificial bones, vascular stents, and cardiac pacemakers) greatly improve the quality of human life.^[^ ^10^, ^11^ ^]^ Moreover, biomaterials gradually play more and more significant roles in cancer treatment.^[^ ^12^, ^13^, ^14^ ^]^ Gels and hydrogels are one of the most widely used biomaterials in postoperative management. They are commonly used in clinics as wound dressings to provide protective barriers against bacteria, suitable environment for wound healing, and relief of pain in the injured site, while they can be detached after healing without tearing wound area owing to their non‐adhesiveness.^[^ ^15^, ^16^, ^17^ ^]^ On the basis of their similarities in structure and composition to natural extracellular matrix (ECM), they have the capability to mimic the environment in native tissue and communicate with cells by biological signals.^[^ ^18^ ^]^ Together with their unique and designable three‐dimensional (3D) structures, they are promising in serving as platforms for drug loading and delivery to meet specific needs from different patients. Recently, researchers have established a series of in situ drug‐loaded biomaterial platforms including gel/hydrogel, aiming at local treatment to inhibit tumor recurrence and metastasis after primary tumor removal surgery. Compared with traditional drugs that are mainly administrated systemically and need to enter the blood circulation before arriving at the tumor sites, the in situ biomaterials are directly applied to the nidus. Through this way, the locally released drugs have much closer distance and higher chance to access the targets. The loaded drugs not only can be released under control in complex extracellular environment to achieve more efficient effects, but also limit the systemic toxicity to distant normal cells.^[^ ^19^ ^]^ Also, the sufficient drug storage inside the biomaterial platforms contributes to long‐term drug releasing, clearing the residual tumor cells as many as possible.^[^ ^20^, ^21^ ^]^

**FIGURE 1:**
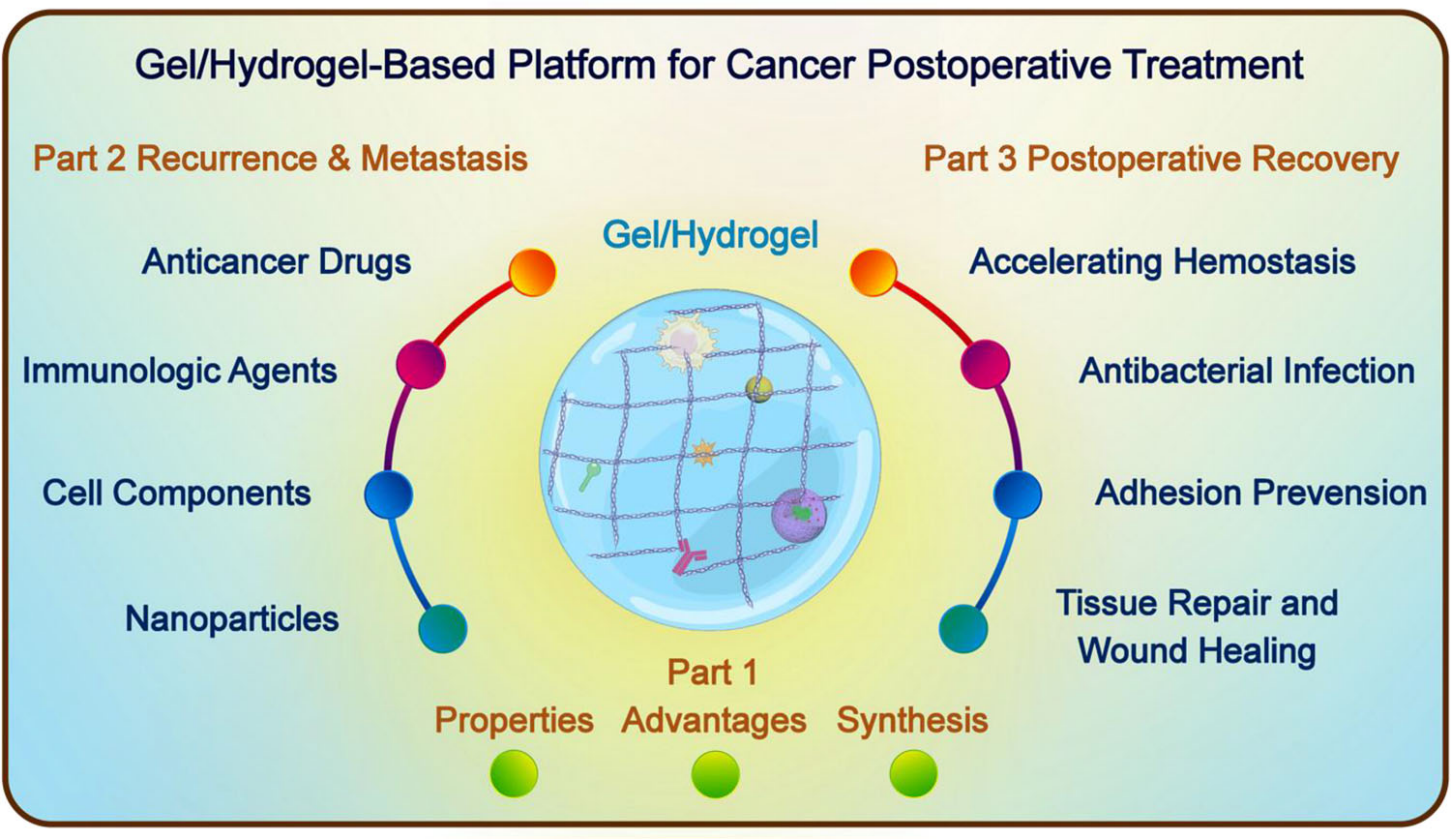
This review focuses on introducing the following three parts. Part 1: The properties, advantages, and synthesis procedures of gel/hydrogel. Part 2: Based on the different types of drugs loaded in the gel/hydrogel, the local delivery system can achieve the inhibition of postoperative tumor recurrence and metastasis. Part 3: Other postoperative applications of gel/hydrogel for disease recovery.

Here, we would like to address the concept of gel/hydrogel‐based in situ biomaterial platforms, which may be promising solutions to overcome the challenges in tumor post‐surgical treatment, such as wound management, cancer relapse, and metastasis. However, few publications focused on the systemic and comprehensive introduction of this concept. Hence, we would like to fill in the literature gap to summarize the characteristics and applications of gel/hydrogel‐based in situ biomaterial platforms, aiming to provide ideas for researchers in this field. In this review, we will start with the introduction of gel and hydrogel by addressing their characteristics and advantages. In the following sections, we elaborate on the current research achievements and their roles in post‐surgical tumor recurrence and metastasis inhibition and discuss the merits and demerits of different kinds based on the types of contents loaded in the platforms. Additionally, we will also introduce the applications of the in situ biomaterial platforms in postoperative recovery process, including accelerating hemostasis, antibacterial infection, adhesion prevention, tissue repair, and wound healing (Figure 1). In the end, we summarized the current challenges of in situ gel/hydrogel platforms development and provided the direction for future research.

## 2 GEL/HYDROGEL CHARACTERISTICS, ADVANTAGES, AND SYNTHESIS PROCEDURES

### 2.1 Gel

Gels are elastic and jelly‐like materials that are composed of 3D cross‐linked polymers immersed in a solvent. This colloidal network may range from soft to rigid depending on the physical and chemical forces between the polymeric strands of the matrix, usually hinging upon different salt concentrations.^[^ ^22^, ^23^ ^]^ These non‐covalent interactions, such as metal coordination, hydrogen bonding, π–π stacking, hydrophobic and van der Waals interactions, make gels soft matters with reversible and stimuli‐responsive characteristics, which are capable to be broken and reformed dynamically under certain stimulation.^[^ ^24^ ^]^ In addition, different functional motifs or drugs can be assembled into gel systems through various processes, indicating that researchers can easily design gels with desired properties and functions.^[^ ^25^, ^26^ ^]^ Besides, with the proper control of gelator concentration, pH, temperature, and other stimulations, the sol‐to‐gel transformation time can be adjusted to an expected period, which allows the in situ gel system establishment on a specific site.^[^ ^27^, ^28^ ^]^ Ishii et al. developed an exenatide‐loaded injectable gel for type 2 diabetes treatment, which was converted from micelles at room temperature to gel under physiological temperature.^[^ ^29^ ^]^ Similar applications of rapid sol‐to‐gel transformation have been applied to treatment of vocal fold (VF) scarring,^[^ ^30^ ^]^ hyperlipidemia,^[^ ^31^ ^]^ bone defect,^[^ ^32^ ^]^ and so forth. Based on these advantages, gels can also serve as promising drug delivery media for post‐surgical in situ anti‐tumor application with condition‐specific controlled release of drugs, achieving high local therapeutic efficacy and low systemic effect compared to traditional chemotherapy drugs.^[^ ^22^ ^]^ To prepare gels, three compositions need to be mixed, including monomer, initiator, and cross‐linker together with diluters for final gel property adjustment. As gels are composed of cross‐linked polymeric networks, various polymerization techniques can be applied to prepare gels, including bulk polymerization, solution polymerization, suspension polymerization, grafting to a support surface, and polymerization by irradiation.^[^ ^33^ ^]^

### 2.2 Hydrogel

Hydrogels are one class of gels that use water as solvent or dispersion medium. Compared to other classes of gels, the water content of hydrogels normally exceeds 90%.^[^ ^34^ ^]^ Previously, hydrogels are defined as 3D polymer networks that have the capability to swell and retain significant amounts of water within their structures, at the same time avoiding dissolving in water.^[^ ^35^ ^]^ Recently, hydrogels have been further defined as multi‐component systems composed of 3D polymer chain networks with water filling the space between macromolecules.^[^ ^33^ ^]^ As one subtype of gels, hydrogels show similar characteristics as gels, such as reversible, stimuli‐responsive, malleable, shape adaptive, and rapidly preparable.^[^ ^36^, ^37^ ^]^ Owing to their 3D polymer networks with convenience for drug loading, hydrogels are extensively used for in vivo drug delivery platforms.^[^ ^38^, ^39^ ^]^ Furthermore, their soft nature and high water content provide similar microenvironment as natural ECM that is suitable for cell growth.^[^ ^40^ ^]^ Their biocompatible properties have been proven and widely used in biomedical applications for sustained local treatment.^[^ ^41^, ^42^, ^43^ ^]^ With the combination of these advantages, hydrogel‐based therapy is becoming a promising option for cancer postoperative treatment to inhibit recurrence and metastasis.^[^ ^44^ ^]^ Based on the types of monomer that make up of the hydrogels, they can be classified into polymer hydrogels and small molecular hydrogels. Polymer hydrogels are one of the most widely studied hydrogels, whose preparation is not complicated. In general, polymer hydrogels are simple elastic structures built by hydrophilic polymeric network cross‐linking. Therefore, any mechanisms of interactions between substances to produce cross‐linked polymers can produce polymer hydrogels, such as physical interactions or chemical reactions.^[^ ^33^ ^]^ Based on the types of cross‐linking, polymer hydrogels can be classified into physically cross‐linked hydrogels and chemically cross‐linked hydrogels. Physically cross‐linked hydrogels are reversible gels without using cross‐linker in their gelation process. Instead, they depend on the physical interactions between different polymer chains to prevent dissolution, including ionic interactions and hydrogen bonding. Xie et al. fabricated an ionic cross‐linked hydrogel by ferric ion and poly(acrylic) acid interaction.^[^ ^45^ ^]^ This property makes the hydrogel synthesis easy, which can be achieved by repeating freeze–thawing and stereo complex formation for physically cross‐linked hydrogels' preparation. Chemically cross‐linked hydrogels are synthesized via chemical reactions with covalent bond generation between polymer chains, which guarantees their stability unless cleaving covalent cross‐link points. When the irreversible covalent bonds are damaged, the integrity and mechanical properties of the hydrogel are irreversibly damaged, which dampen the functions and service life of it. However, polymer hydrogels obtained by cross‐linking with reversible chemical bonds, such as Schiff bases, exhibit self‐healing properties and stimuli responsiveness.^[^ ^46^, ^47^ ^]^ Zhang et al. prepared a multiresponsive (e.g., pH, amino acids, and vitamin B6 derivatives) and self‐healing chitosan‐based hydrogel by forming Schiff base linkages between poly(ethylene glycol) and chitosan.^[^ ^48^ ^]^ Many methods can be applied to prepare chemically cross‐linked hydrogels such as chemical cross‐linking, chemical and radiation grafting, radical polymerization, condensation reaction, enzyme reaction, and high‐energy radiation.^[^ ^49^, ^50^ ^]^ Small molecular hydrogels, also known as supramolecular hydrogels, are composed of low molecular weight gelator with less than 2000 Daltons, including nucleic acid derivatives, amino acids, small peptides, carbohydrates, and so forth. Compared to polymer hydrogels, small molecular hydrogels have better biodegradability and biocompatibility, and because of the convenience of design and fabrication, they are attracting increasing attention in the field of drug delivery and cancer therapy.^[^ ^51^ ^]^

## 3 GEL/HYDROGEL REALIZED THE IN SITU POSTOPERATIVE ADJUVANT ANTICANCER THERAPIES

Gels and hydrogels are promising in situ biomaterial platforms for drug delivery in local treatment.^[^ ^52^ ^]^ In cancer postoperative treatment, different types of drugs have been used for different adjuvant therapies. Chemotherapy is one of the most commonly used therapies in clinics, which utilizes chemical drugs to kill the cancer cells or to arrest the cancer cells in their quiescent stage.^[^ ^53^ ^]^ Immunotherapy is another option that kills the cancer cells by stimulating the immune system, using immune activators such as cytokines, vaccinations, and immune checkpoint inhibitors.^[^ ^54^ ^]^ In addition, cell components including DNAs, RNAs, proteins, lipids, cell membranes, or even the whole cells can also be used as the adjuvant to suppress tumor recurrence.^[^ ^55^, ^56^, ^57^ ^]^ Last but not least, nanoparticles have been increasingly used as effective and powerful carriers for anticancer agents with the combination of sensitizers for photodynamic therapy, thermodynamic therapy, sonodynamic therapy or radiotherapy, etc., achieving promising anticancer therapeutic effect.^[^ ^58^ ^]^ All the above mentioned anticancer strategies can be realized in the gel/hydrogel platforms for synergic postoperative tumor recurrence and metastasis inhibition via in situ drug delivery.

### 3.1 Gel postoperative application

#### 3.1.1 The loading of anticancer drugs

In the past decades, the number of cancer patients who received anticancer drugs treatment or chemotherapy has been increasing. Through the mechanisms of cytotoxicity, anticancer drugs can be classified into several categories, including DNA‐interactive agents, antimetabolites, antitubulin agents, and so on. The anticancer drugs take effect by destroying tumor cells or at least suppress cell growth in human body, therefore they are often used to clear the residual tumor cells after surgical resection. With repeated exposure to anticancer drugs, cancer patients may have short‐term tumor suppression effect. However, the side effects caused by chemotherapy such as bone marrow suppression, nausea, and hair loss are non‐negligible.^[^ ^59^ ^]^ On the one hand, long‐term or overdosed chemical drugs can lead to more severe adverse effects such as drug resistance and cardiotoxicity.^[^ ^60^ ^]^ These side effects should be imputed to the indiscriminate attack of anticancer drugs, not only attacking tumor cells but also damaging healthy cells that lead to normal tissue dysfunction because they are usually administrated systemically, traveling through the bloodstream before getting to the tumor sites. On the other hand, incomplete clearance of residual tumor cells becomes the potential risk of post‐surgical recurrence and metastasis, leading to the decreased long‐term survival rate of cancer patients. Hence, developing new strategies for postoperative anticancer drug delivery is an important issue.

**FIGURE 2:**
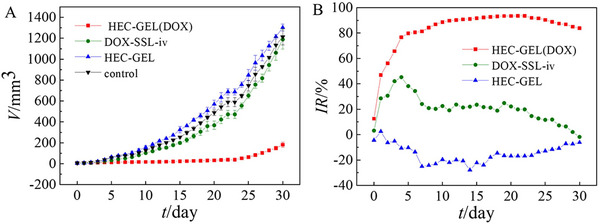
Hydroxyethyl cellulose hemostatic gauze contain doxorubicin‐loaded in situ gel (HEC‐GEL (DOX)) exhibited significant anti‐tumor effect in in vivo evaluation. (A) Tumor volume monitor for in vivo anti‐tumor evaluation. (B) Comparison of tumor inhibition rate in different treatment groups. Reproduced with permission from reference.^[^ ^61^ ^]^ Copyright 2020, American Chemical Society.

With the capabilities of local delivery, controlled release, and sustained supply of the loaded substances, the in situ gel platforms have great potential to be a good alternative to avoid systemic side effects from anticancer drugs. For example, Xie's group loaded the doxorubicin (DOX), one of the most widely used anthracyclines in clinics, in a thermosensitive gel (GEL(DOX)) with the combination of hydroxyethyl cellulose (HEC) gauze (HEC‐GEL(DOX)) for breast cancer postoperative treatment.^[^ ^61^ ^]^ Based on the thermosensitive property, the pre‐mixed DOX and sol would rapidly process gelation at the administration site, which simultaneously helped the fixation of HEC gauze. The HEC gauze is a biodegradable material that can stop the bleeding and prevent the inflammation of the operative incisions, accompanied by the continuous supply of the DOX from gel to eliminate the residual tumor cells. The in vivo postoperative anti‐tumor evaluation manifested the superior anti‐tumor efficacy of the combination of HEC‐GEL(DOX), whose tumor inhibition rate was twice as high as traditional systemically administrated DOX (Figure 2). These results proved that compared to systemically administrated anticancer drugs, the in situ gel platform not only efficiently prevented tumor recurrence and metastasis after surgical resection but also limited the cytotoxicity to normal tissues. Similarly, other anticancer drugs with disappointed side effects such as cisplatin‐triggered nephrotoxicity and bleomycin‐triggered pulmonary fibrosis may also be delivered in the way of in situ gel platforms for a better therapeutic effect and a reduced adverse effect.^[^ ^62^, ^63^, ^64^ ^]^ For more complicated or drug‐resistant cancers, immunologic agents combined with chemical drugs to modulate tumor immune microenvironment can be a potential solution.^[^ ^65^ ^]^

#### 3.1.2 The loading of immunologic agents

**FIGURE 3:**
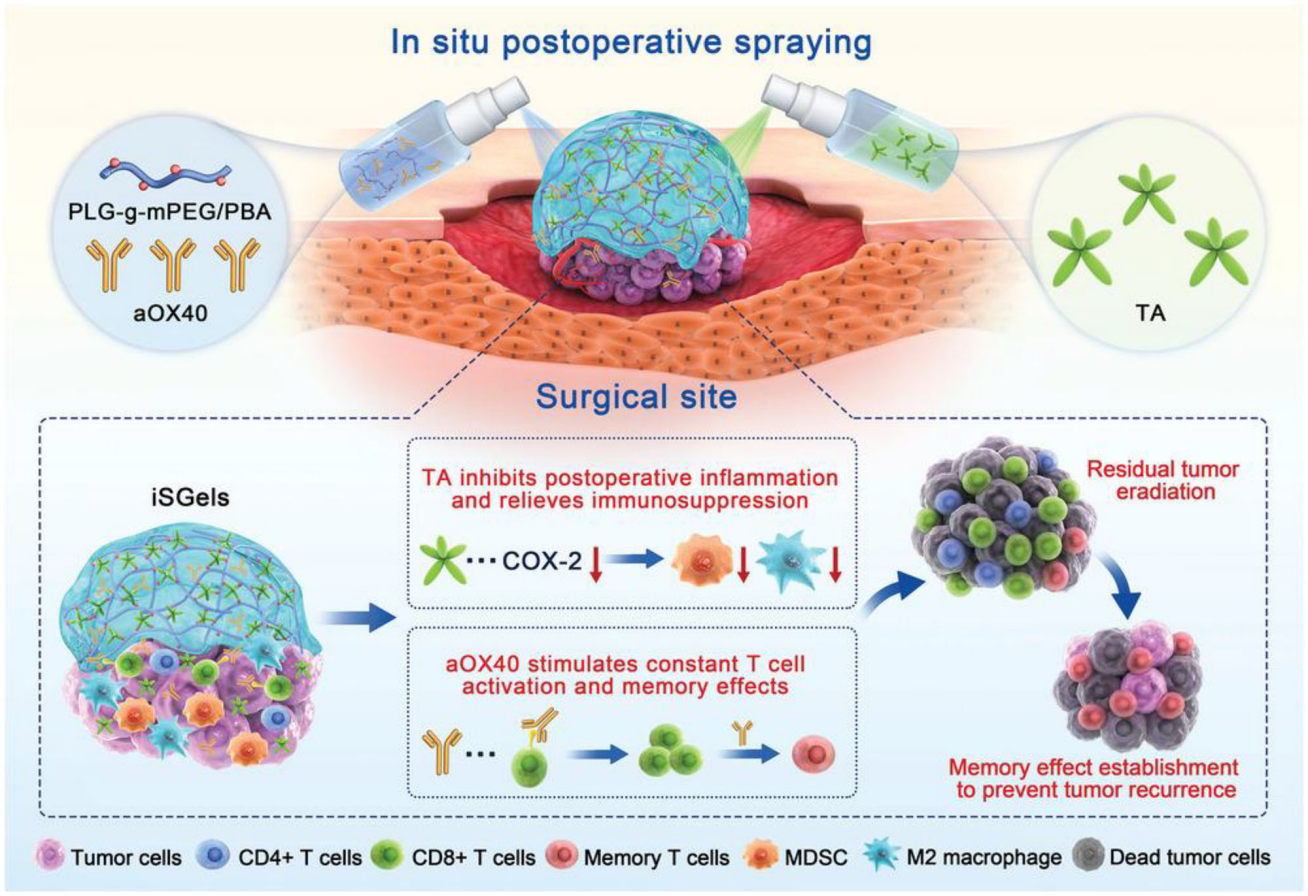
Design of in situ sprayed immunotherapeutic gel loaded with anti‐OX40 antibody (iSGels@aOX40) for colorectal cancer post‐surgical treatment. COX‐2, cyclo‐oxygenase‐2, an inflammatory mediator enzyme. Reproduced with permission from reference.^[^ ^73^ ^]^ Copyright 2021, Wiley‐VCH GmbH.

In recent years, as tumor resection‐induced immunosuppression may accelerate post‐surgical local recurrence and distant metastasis, immunotherapy has become a powerful strategy in cancer treatment.^[^ ^66^, ^67^ ^]^ Compared with traditional chemotherapy that acts by directly killing the tumor cells, immunotherapy focuses on activating immune system and improving anti‐tumor immune responses, which have great potential to be the solution to target on immune escaping tumor cells.^[^ ^68^ ^]^ Current immunotherapy can be divided into immune checkpoint blockade therapy, engineered T cell adoptive immunotherapy, cytokines‐promoted lymphocytes stimulation, tumor‐specific antigens and receptors targeting, and cancer vaccines.^[^ ^69^ ^]^ Different classes depend on different immune agents, such as CTLA‐4 and PD‐1 antibody for immune checkpoint blockade therapy, chimeric antigen receptor T cells (CAR‐T cells) for T cell adoptive immunotherapy, and interferons for cytokines‐promoted lymphocytes stimulation. However, traditional administration of these immunologic agents may lead to low response rate and systemic immune‐related adverse effect.^[^ ^70^ ^]^ These unfavorable outcomes can be improved by loading immunologic agents into biomaterial platforms to construct local drug delivery system.^[^ ^71^, ^72^ ^]^ For example, Si et al. developed an in situ immunotherapeutic gel for postoperative colorectal cancer (CRC) treatment.^[^ ^73^ ^]^ Although most advanced CRC patients received surgical resection as the main treatment, the poor prognosis together with the high recurrence and metastasis rate disappointed them a lot. It is stimulated by the postoperative wound‐caused immunosuppressive environment with classic characterizations of inflammation such as neutrophile granulocytes infiltration and proinflammatory cytokines release.^[^ ^74^, ^75^ ^]^ Hence, they loaded the immune agonistic anti‐OX40 (aOX40) antibody in the in situ sprayed immunotherapeutic gel (iSGels@aOX40 gel), which was composed of the tannic acid (TA) and poly(l‐glutamic acid)‐g‐methoxy poly(ethylene glycol)/phenyl boronic acid (PLG‐g‐mPEG/PBA). The combination of the anti‐inflammatory TA and the aOX40 proposed to enhance the effector T cell activation and suppress the regulatory T cells (Tregs) significantly induced immunogenic cell death, leading to an improvement of the prognosis by modulating the tumor microenvironment (TME) (Figure 3). Besides, compared to intravenous injection, the in situ formed spraying gels can not only fit into the irregular shape of the wounds and provide a sustained supply of drugs directly to the residual cells, but also prevent self‐inactivation and degradation of active ingredients by the enzymes in the environment. Therefore, the maximum anti‐recurrence effect of immunotherapy can be realized through this delivery platform.

#### 3.1.3 The loading of cell components

**FIGURE 4:**
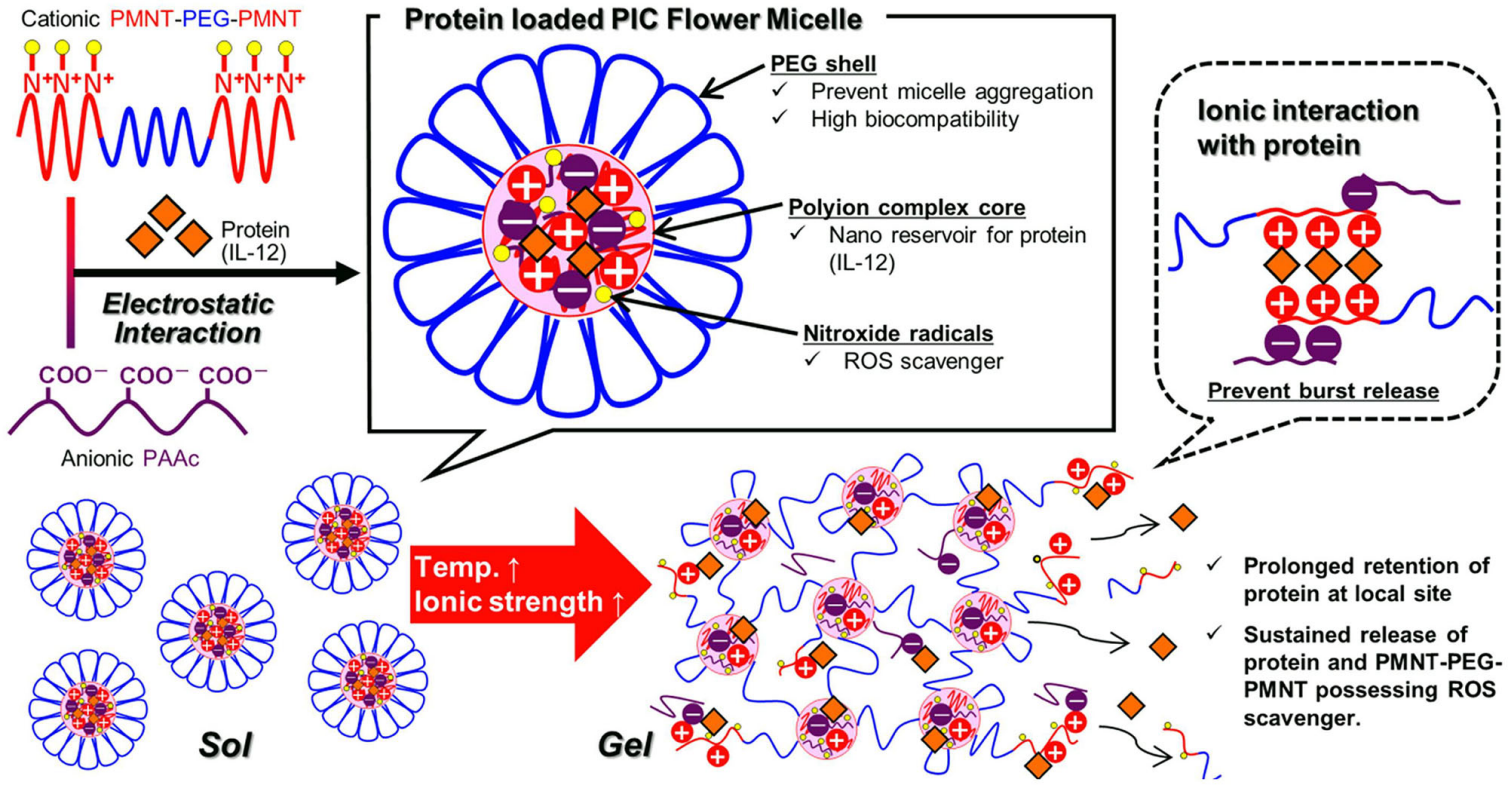
Schematic design of polyion complex flower micelle and the functions and properties of redox‐active, injectable, gel. Reproduced with permission from reference.^[^ ^77^ ^]^ Copyright 2016, Elsevier Ltd.

In recent years, researchers have been exploring the potential of cell components including DNAs, RNAs, and proteins to serve as therapeutic agents for cancer treatment because of their vital roles in biological processes. However, the delivery efficacy and the releasing rate still need to be controlled in a better manner.^[^ ^76^ ^]^ Nagasaki's group developed an in situ biomaterial platform named redox‐active, injectable, gel (RIG) to realize the local delivery of bioactive proteins in the TME with a suitable release rate to avoid excessing the maximum tolerable dose, maintaining the best therapeutic concentration of interleukin‐12 for tumor suppression.^[^ ^77^ ^]^ The polymer used to construct the network was the poly[4‐(2,2,6,6‐tetramethylpiperidine‐*N*‐oxyl)aminomethylstyrene]‐*b*‐poly(ethylene glycol)‐*b*‐poly[4‐(2,2,6,6‐tetramethylpiperidine‐*N*‐oxyl)aminomethylstyrene] PMNT‐PEG‐PMNT triblock copolymer that can form polyion complex (PIC) flower micelle, whose electrostatic interaction can help the encapsulation of the charged substances such as proteins, and simultaneously the rapid diffusion would be limited in a suitable speed. Additionally, the side chain of the PMNT segment would catalyze the elimination of the reactive oxygen species (ROS) by covalent bonds formation, hence relieving the ROS‐induced oxidative stress that could induce inflammation. The application of the RIG to the tumor sites is also convenient and easy to use. The protein‐mixed PIC flower micelle solution showed the property of sol‐to‐gel irreversible transition when heating to physiological temperature, which allowed rapid in situ formation (Figure 4). This protein‐loaded in situ formed multifunctional gel system skillfully utilized the advantages of biomaterial to create a more effective and prolonged delivery platform with fewer adverse effects, enlightening a new method to deliver other cell components and biomolecules with therapeutic aims.

#### 3.1.4 The loading of nanoparticles

Assembly of different functional components into nanoparticles is a promising method to congregate complicated functions in one platform. Therefore, the combination of nanoparticles and in situ biomaterials for local delivery is a hot spot in recent cancer postoperative treatment. For example, Chen et al. developed an anti‐CD47 antibody‐loaded CaCO_3_ nanoparticle (aCD47@CaCO_3_) containing bio‐responsive gel combined with immunotherapy to suppress tumor recurrence.^[^ ^78^ ^]^ They synthesized a fibrinogen and thrombin co‐formed fibrin gel as the delivery platform of nanoparticles, which is biocompatible and sprayable to cover the injured tissues and promote wound healing. The loaded CaCO_3_ nanoparticles could be released continuously and reacted with the acidic substance in the TME, leading to the acid‐responsive specific release of aCD47 in the acidic TME, at the same time serving as the proton scavenger to modulate the immunosuppressive TME. In this scenario, the released aCD47 blocked the interaction between CD47 and SIPRα, inhibiting the macrophage phagocytosis. Compared to the systemic administration of nanoparticles, which is difficult to target and accumulate at tumor sites and the majority of nanoparticles still accumulate in the liver, the local administration of nanoparticles with the assistance of gel platform significantly enhanced the delivery efficacy and targeted capability. In addition, the combination boosted the anti‐tumor T cell response locally to eliminate the residual tumor cells, which prevented the tumor recurrence and metastasis and avoided severe systemic effects. Apart from immunotherapy, other traditional therapies can also be realized in the in situ biomaterial platforms. Qin et al. designed an α‐cyclodextrin‐based gel system loaded with a multifunctional nanoparticle composed of photothermal agent, indocyanine green, chemotherapeutic drug DOX, and an immunomodulator CpG, together as a cancer vaccine to counter the postoperative tumor recurrence and metastasis.^[^ ^79^ ^]^ Similar therapies such as sonodynamic therapy,^[^ ^80^ ^]^ chemodynamic therapy,^[^ ^81^ ^]^ and chemophotothermal therapy,^[^ ^82^ ^]^ have also been combined into in situ biomaterial platforms and achieved great success.

### 3.2 Hydrogel post‐surgery application

#### 3.2.1 The loading of anticancer drugs

**FIGURE 5:**
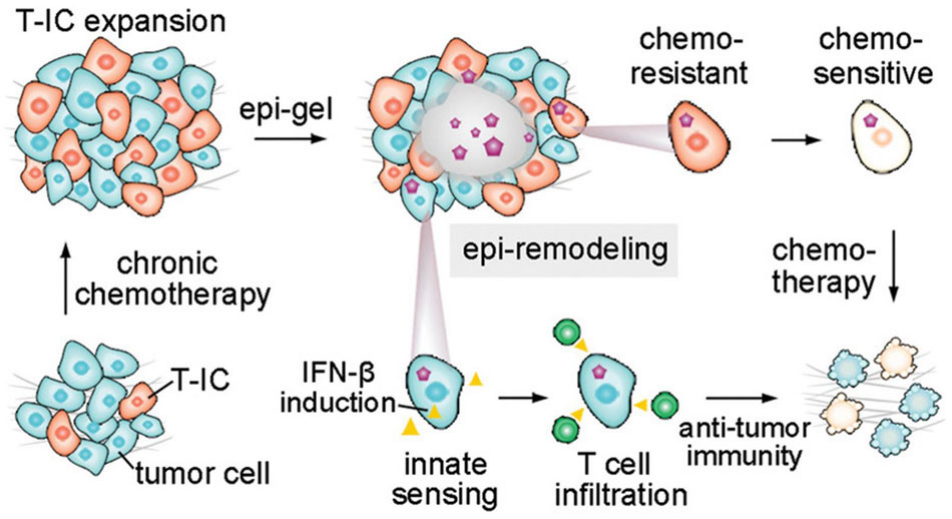
Schematic representation of the functions of epi‐gel in chemoresistant triple‐negative breast cancer. Reproduced with permission from reference.^[^ ^84^ ^]^ Copyright 2021, Wiley‐VCH GmbH.

**FIGURE 6:**
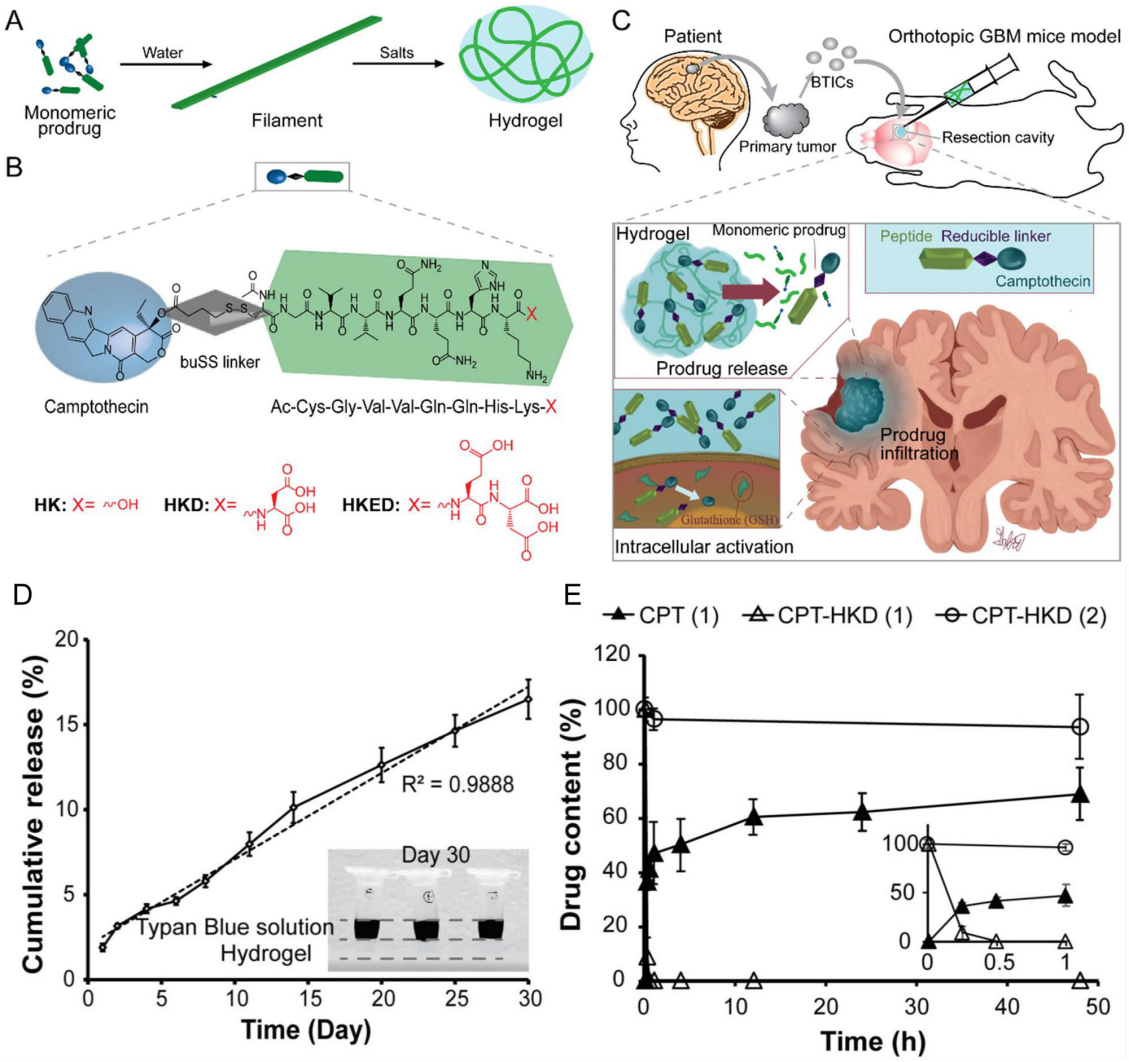
Design and characterizations of prodrug hydrogel. (A) Schematic illustration of self‐assembly process from monomeric prodrug to hydrogel. (B) Chemical structures of camptothecin (CPT) prodrug. (C) The intended use of self‐assembling prodrug hydrogel for brain tumor local treatment. (D) Sustained release of camptothecin‐buSS‐CGV2Q2HKD‐OH (CPT‐HKD) from hydrogel in 30 days. (E) CPT‐HKD can effectively convert into CPT in the presence of glutathione (1). In contrast, CPT‐HKD cannot effectively convert into CPT in the absence of glutathione (2). Reproduced with permission from reference.^[^ ^87^ ^]^ Copyright 2020, Elsevier B.V.

As an efficient drug delivery platform, hydrogel is widely utilized to carry anticancer drugs for postoperative applications.^[^ ^83^ ^]^ Song's group reported an epigenetic drugs harboring hydrogel to improve chemoresistance and metastasis in triple‐negative breast cancer (TNBC) patients.^[^ ^84^ ^]^ On account of lacking the expression of three principal receptors (HER2, human epidermal growth factor receptor; PR, progesterone receptor; ER, estrogen receptor) as the major medication target, TNBC patients are obliged to rely on traditional chemotherapy. However, repeated chemotherapeutic treatment stimulates the proliferation of tumor‐initiating cells (T‐ICs) in tumor residue, which have great capability of self‐renew and resistance to multiple drugs, leading to decreased chemotherapeutic effect and increased tumorigenesis.^[^ ^85^ ^]^ To solve this problem, they developed an epigenetic therapy by loading lysine‐specific demethylase 1 (LSD1) inhibitor in the hydrogel to improve the chemosensitivity of drug‐resistant tumors, named as epigenetic hydrogel (epi‐gel). LSD1 regulated the cancer cell self‐renewal and differentiation by catalyzing demethylation of histone H3 lysine 4. When the function of LSD1 is inhibited, the tumor stem cells will initiate differentiation and loss the stemness maintenance as the result of epigenetic regulation, which is beneficial to increase chemosensitivity and reduce drug resistance, and further prevent postoperative tumor recurrence and metastasis (Figure 5). Additionally, traditional chemical drugs used in clinic can also be loaded inside hydrogel platforms to achieve promising recurrence‐prevention therapeutic effect, such as gemcitabine (GEM)^[^ ^86^ ^]^ and DOX^[^ ^36^ ^]^. Apart from anticancer drugs, prodrugs can also be delivered through hydrogel platforms to realize local treatment with high efficiency. Cui and his group reported their work on developing a self‐assembling prodrugs (SAPD) hydrogel composed of camptothecin (CPT)‐buSS‐CGV2Q2HKD‐OH (CPT‐HKD) as a new strategy for glioblastoma multiforme (GBM) postoperative treatment (Figure 6A,B).^[^ ^87^ ^]^ CPT is a topoisomerase inhibitor which is found to be an effective parent drug for GBM treatment.^[^ ^88^ ^]^ In the form of hydrogel, they realized CPT‐HKD in situ administration in a prolonged period of time to eliminate remaining brain tumor‐initiating cells (BTICs) after surgery (Figure 6C). As shown in Figure 6D,E, compared to traditional systemic chemotherapy, hydrogel platform shows distinct advantages in avoiding the deducted delivery efficacy limited by blood–brain barrier (BBB), boosting therapeutic effect by continuous drug release to brain parenchyma, suppressing tumor recurrence and prolonging survival for patients.

#### 3.2.2 The loading of immunological agents

Cancer immunotherapy has become a promising option for the prevention of postoperative tumor recurrence and metastasis with efficient therapeutic effect. Many researchers have tried to apply this strategy in local treatment to avoid severe systemic side effect by loading immunologic agents in hydrogel platforms for delivery. Zhang et al. designed a fibrin hydrogel as a therapeutic scaffold to co‐deliver cyclophosphamide (CTX) and anti‐PD‐L1 antibody (aPDL1) to regulate TME.^[^ ^89^ ^]^ CTX can not only induce immunogenic apoptosis of tumor cells by calreticulin upregulation but also strengthen the anti‐tumor effect of cytotoxic T lymphocytes by limiting the frequency of Tregs in TME. With the premodification of TME by CTX, the later released aPDL1 and achieved a maximized therapeutic efficacy in immune checkpoint blockade therapy. Therefore, the synergistic anticancer effect significantly reversed the immunosuppressive TME and greatly decreased the risk of postoperative cancer recurrence and metastasis. Similar immunologic agents can also be combined and embedded into hydrogels to regulate TME and enhance immune response to suppress tumor recurrence, including toll‐like receptor agonists,^[^ ^90^ ^]^ immunoadjuvant CpG oligonucleotide,^[^ ^91^ ^]^ and granulocyte–macrophage colony stimulating factor.^[^ ^92^ ^]^ When dying cancer cells, tumor lysates, or treated dendritic cells are encapsulated inside the hydrogels, cancer vaccines can be applied to the tumor sites to induce the suppression of local tumor recurrence and distant metastasis.^[^ ^93^, ^94^, ^95^ ^]^

#### 3.2.3 The loading of cell components

Cell components can be utilized as the composition of in situ drug delivery biomaterial platform. For instance, apart from the ability to encode genetic information, DNA has been used for the construction of hydrogels via sequence‐directed hybridization because of its programmable assembly capability and biocompatibility.^[^ ^96^, ^97^ ^]^ Based on their previous experience, Zhang's group modified the DNA‐constructed drug delivery hydrogel with higher drug molecules loading amount.^[^ ^98^ ^]^ They grafted the chemical drug CPT onto the backbone of the phosphorothioate oligonucleotide, achieving conjugation between drugs and DNA without affecting base pairing and sequence‐dependent assembly. Next, the obtained CPT‐grafted DNAs were constructed into Y‐shaped motifs and then assembled into hydrogel networks via “sticky‐end” association.^[^ ^99^ ^]^ This DNA‐loaded in situ hydrogel platform not only maintained the reversible and injectable capability of the pristine hydrogel with high drug loading capability but also showed excellent biocompatibility, and efficient penetration in the tumor sites by enzymatic degradation‐dependent disassembly. In addition, other cell components can also be conjugated into hydrogel networks to inhibit tumor recurrence and metastasis by local treatment, such as RNAs,^[^ ^100^, ^101^ ^]^ proteins,^[^ ^102^, ^103^, ^104^ ^]^ enzymes,^[^ ^105^, ^106^ ^]^ and liposomes.^[^ ^21^, ^107^, ^108^ ^]^ Recently, researchers developed an exogenous cell‐embedded hydrogel that achieved ideal tumor postoperative relapse inhibition. Hu and his teammates successfully encapsulated CAR‐T cells that target on human chondroitin sulfate proteoglycan 4 (CSPG4), and aPDL1 antibody conjugated platelets into the hydrogel reservoir.^[^ ^109^ ^]^ CSPG4 is a specific marker with significantly higher expression in human melanoma cells than in normal cells, which is a target for CAR‐T cells to recognize and eradicate residual melanoma. This combination of immunotherapy skillfully utilized the postoperative inflammation that could induce tumor recurrence and metastasis to activate the platelets inside the hydrogels, triggering the release of aPDL1 antibodies to block the immune checkpoint pathways, and preventing tumor recurrence. Furthermore, Wang claimed that the treated cancer cells could be loaded in peptide hydrogels as personalized cancer vaccines in postoperative applications, combined with photodynamic therapy, it may become a novel strategy for postoperative immunotherapy.^[^ ^110^ ^]^ In summary, hydrogel platforms serve as the deliverer and protector for cell components. On the one hand, they provide a barrier for cell components that are against enzyme degradation in the physiological environment. On the other hand, hydrogel platforms deliver the cell components to the target site and continuously release them, which efficiently elevate the response rate of immunotherapy and avert cancer recurrence and metastasis.

#### 3.2.4 The loading of nanoparticles

In cancer treatment, nanoparticle technology has been proved to have promising advantages in drug delivery, such as high drug loading yield, condition‐controlled release, and targeted delivery. Many nanoparticles with significantly improved therapeutic index of drugs have been developed to treat various types of diseases, aiming to achieve better therapeutic effects in advanced nanomedicine.^[^ ^111^ ^]^ Meanwhile, the formation of hybrid systems combining nanoparticles with hydrogels is receiving more and more attention.^[^ ^112^, ^113^, ^114^ ^]^ Zhang's group named these systems as the nanoparticle‐hydrogel hybrid systems (NP‐gels) and concluded three roles of nanoparticles in the hydrogel network formation process.^[^ ^112^ ^]^ First, nanoparticles can be one composition of hydrogel by mixing with monomer solution, and after gelation they will be embedded into the network. Second, nanoparticles can be directly “uptaken” by swelling hydrogel for incorporation, through which the gelation process will not be interfered by nanoparticles. With proper adjustment of compositions, the hydrogel network can protect the structures and maintain the functions of the inside nanoparticles. Third, nanoparticles can also act as the cross‐linkers contributing to 3D hydrogel network construction. They can be linked together via covalent interactions, strong hydrophobic interactions, or opposite charge attractions to acquire hydrogel‐like properties. Compared with only nanoparticles, NP‐gels hybrid systems integrate the strengths of two types of materials, achieving strong synergistic effect in drug delivery.

**FIGURE 7:**
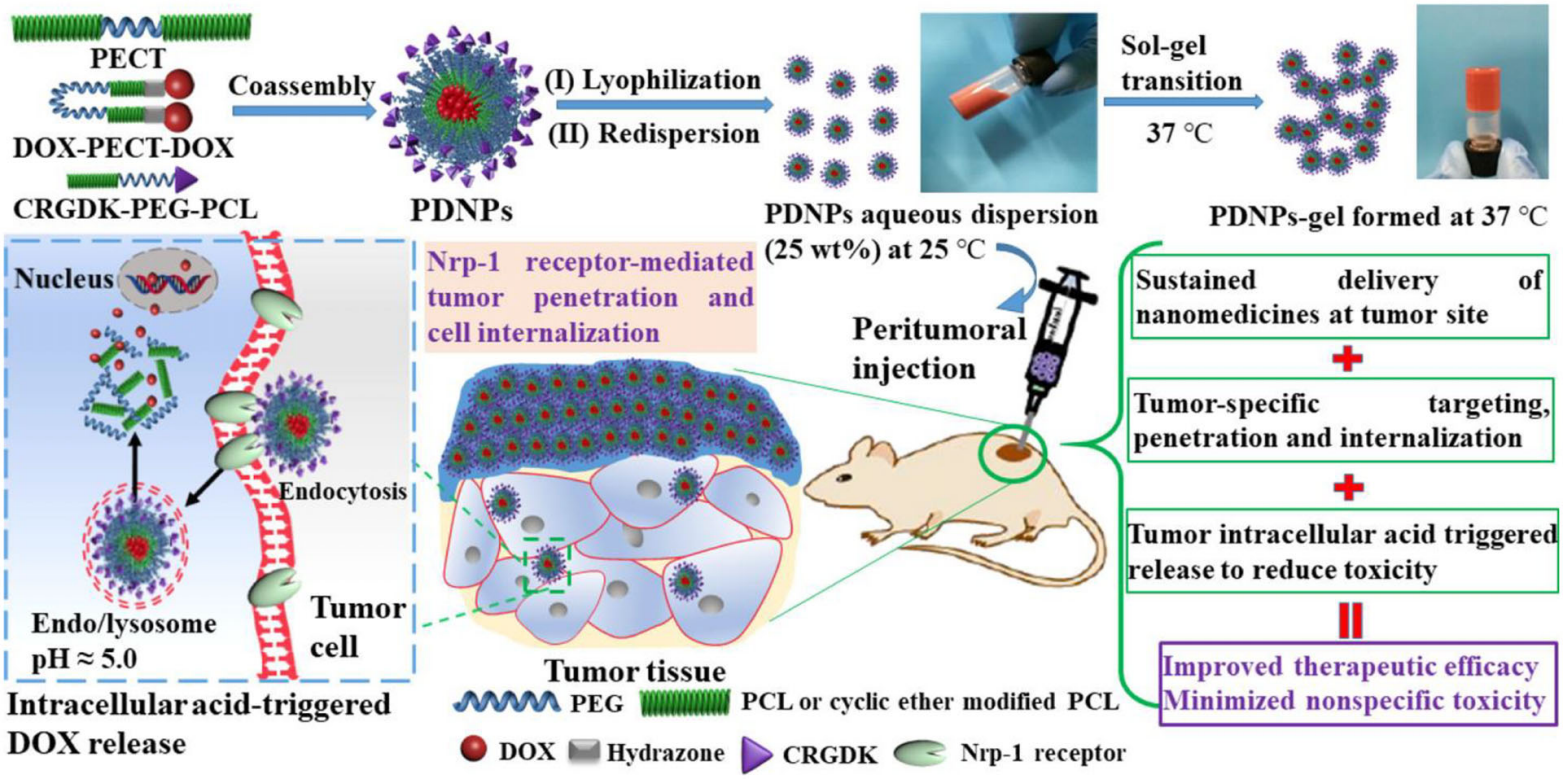
Schematic illustration of synthetic process and physiological functions of CRGDK (Cys‐Arg‐Gly‐Asp‐Lys)‐mediated hydrogel‐embedded nanoparticles. Reproduced with permission from reference.^[^ ^120^ ^]^ Copyright 2019, American Chemical Society.

Taking advantage of this biomaterial platform, researchers have been trying to design NP‐gels with specific properties and desired functions for cancer post‐surgery applications, especially for “stubborn” cancers. GBM is the most aggressive type of brain tumor with high proliferation activity and high tumor cell infiltration rate into surrounding brain parenchyma, which is hard to be completely removed by surgical resection. To make things worse, the BBB preventing most drug from entering the brain and the long period for wound healing leads to untimely radio/chemotherapy regimen which is essential for killing residual infiltrated tumor cells. Therefore, GBM patients have to face up to poor prognosis and tumor recurrence. To prevent postoperative GBM recurrence, Preat's group developed a hydrogel loaded with cytotoxic paclitaxel‐poly lactic‐*co*‐glycolic acid (PLGA) nanoparticles as an in situ drug delivery system, which can not only contact tumor cells with drugs to reduce off‐target effects but also maintain sustained drug supply and limit drug degradation.^[^ ^115^ ^]^ As the result showed, this NP‐gels hybrid system achieved significant tumor recurrence suppression effect, which is nearly impossible for traditional therapies or merely nanoparticles. More than that, the NP‐gels hybrid system achieved specific therapeutic effects that systemically administrated nanoparticles cannot accomplish in other types of cancer, such as breast cancer,^[^ ^83^, ^116^ ^]^ lung cancer^[^ ^117^ ^]^, liver cancer,^[^ ^118^ ^]^ and uveal melanoma^[^ ^119^ ^]^. Nevertheless, Zhang's group pointed out that even the combination of nanoparticles and hydrogel showed some advantages for local treatment, there were still some challenges in translational medicine that need to be overcome.^[^ ^120^ ^]^ Because of the higher interstitial fluid pressure and the higher drug concentration gradient in tumors than surrounding tissues, random drug diffusion into normal tissues limited the efficacy of drug delivery. Therefore, they modified the hydrogel‐embedded nanoparticles with a tumor‐specific peptide CRGDK (Cys‐Arg‐Gly‐Asp‐Lys) that targeted on neuropilin‐1, which is a transmembrane receptor glycoprotein overexpressed in tumor site (Figure 7). This ligand receptor‐specific recognition effectively enhanced the internalization of nanoparticles into residual tumors and prevent tumor recurrence, which may promote the progress of research results to the clinical translation.

In summary, in situ gel/hydrogel platforms serve as ideal delivery systems in cancer postoperative treatment, which are promising materials for future clinical translation. Some representative examples are summarized in Table 1. First, the 3D structure of gels/hydrogels endows them with considerable loading capacity and retention ability. The loading components can maintain their structures and activities under the protection of the platforms until they are released. This can avoid frequent injection of drugs compared to traditional chemotherapy or immunotherapy. Second, the components are directly released at the target site without circulating in the bloodstream. With the help of in situ platform, the majority of drugs can perform local anti‐tumor effect. On the one hand, the therapeutic effect is increased and the rate of tumor postoperative recurrence and metastasis is decreased. On the other hand, systemic toxicity is significantly reduced, which is beneficial for healthy tissues and organs. Third, the in situ gel/hydrogel platforms can be flexibly designed with desired functions or multifunction that can fulfill personalized requirements according to their situations. However, the design and the synthesis routine of multifunctional gel/hydrogel platforms are usually complicated and expensive, which makes it difficult to achieve mass production at low cost. In addition, the functionalized platforms are designed for specific patients denoting that they cannot be widely prescribed to patients even with the same cancer, which also limits the use and increases the cost.

**TABLE 1:** Summary of representative gel/hydrogel biomaterial platforms examples including their names, loading components, cancer models that are used to test their therapeutic effects, anticancer therapies, and their advantages. They are classified into four categories based on the components they loaded, including anticancer drug loaded, immunologic agents loaded, cell components loaded, and nanoparticles loaded

Category	Name	Loading components	Cancer	Anticancer therapy	Advantages	References
Anticancer drug loaded	HEC‐GEL(DOX)	Doxorubicin (DOX)	Breast cancer	Chemotherapy	Rapid hemostasis; Retention and sustained release of drugs; Thermosensitive; Low systemic toxicity	[61]
	D/T gel	DOX and resatorvid (TAK‐242)	Breast cancer	Chemoimmunotherapy	Increased accumulation at tumor site; Low systemic toxicity; Retention and sustained release of drugs; Pro‐healing capacity; Reduced inflammation	[65]
	iLSD1@Gel	Lysine‐specific demethylase 1 inhibitor (iLSD1)	Triple‐negative breast cancer	Chemotherapy	ROS responsive; Trigger chemoresistant tumor to chemosensitive tumor	[84]
	CaCO_3_ and PLA‐modified 3D‐printed hydrogel	Gemcitabine (GEM)	Pancreatic cancer	Chemotherapy	Prolonged release of drug; Local delivery of drug	[86]
	GD‐HA/CS‐Gel	GEM and DOX	Breast cancer	Chemotherapy	Injectable; Controllable drug release; Low systemic toxicity	[36]
	Self‐assembling prodrug (SAPD) hydrogel	Camptothecin (CPT)‐buSS‐CGV2Q2HKD‐OH (CPT‐HKD)	Glioblastoma	Chemotherapy	Retention and sustained release of drugs; Efficiently deliver drugs across blood–brain barrier (BBB)	[87]
Immunologic agents loaded	iSGels@aOX40 gel	Anti‐OX40 antibody	Colorectal cancer	Immunotherapy	Sprayable; Antioxidant; Anti‐inflammatory; Antimicrobial; Retention and sustained release of drugs	[73]
	Fibrin hydrogel	Cyclophosphamide (CTX) and anti‐PD‐L1 antibody (aPDL1)	Breast cancer	Chemoimmunotherapy	Sequential local co‐delivery; Low systemic toxicity	[89]
	ICG@SA‐aPDL1 nanogel	CpG ODNs and carcinoembryonic antigen (CEA) probe	Breast cancer	Immunotherapy	Monitor tumor development and recurrence; Protect therapeutic agents from degradation; Strengthen delivery to immune cells; Sustained release of drugs	[90]
	ALG‐Aapt/CpG smart hydrogel	CpG ODNs	Colon cancer	Immunotherapy; Chemotherapy; Radiotherapy	ATP responsive; Controllable release of immune adjuvants	[91]
	mPEG‐b‐poly(l‐alanine) hydrogel	CTLA‐4, PD‐1, tumor cell lysates (TCLs) and granulocyte–macrophage colony‐stimulating factor (GM‐CSF)	Melanoma	Immunotherapy	Protect antigens from degradation; Local and sustained delivery of cancer vaccine	[92]
Cell components loaded	CSSH/Cur‐Lip gel	Liposomes and curcumin	Breast cancer	/	Injectable; Thermosensitive; Local accurate and sustained drug delivery	[21]
	Protein@RIG	Bovine serum albumin (BSA); IL‐12	Colon cancer	Immunotherapy	Injectable; Protect proteins from degradation; Retention and sustained release of proteins; ROS scavenging	[77]
	CPT–DNA‐Gel	DNA and CPT	Colon cancer	Chemotherapy	Injectable; Negligible systemic toxicity; Retention and sustained release of drugs	[98]
	RNA hydrogel	DNA CpG, shRNA, MnO2@Ce6, and DOX	Triple‐negative breast cancer	Chemo‐photodynamic therapy; Gene therapy	Enhanced gene targeted capability; On‐demanding drug release; Low cytotoxicity	[100]
	GLP‐R848‐mOVA Gel	Resiquimod (R848) and ovalbumin encoding mRNA (mOVA)	Melanoma	Immunotherapy	Injectable; Enhanced and precise delivery capability; Sustained release of RNA vaccine; Protect mRNA from degradation	[101]
	PAA‐BB@Ricin/Tf	Ricin and transferrin protein (Tf)	Liver cancer	/	Enhanced tumor targeted capability; High loading efficiency	[102]
	CytoC/aNGs/Gel	Cytochrome c	Breast cancer	/	Injectable; Enhanced delivery capability; Protect proteins from degradation	[105]
	CAG hydrogel	Glucose oxidase	Breast cancer	Photothermal therapy	Injectable; pH‐sensitive photothermal conversion property; Low toxicity; Low cost	[106]
	CSSH/Cur@Lip‐Cos‐PC	Liposomes and curcumin	Breast cancer	/	Injectable; Minimized damage to healthy non‐target tissues; Enhanced delivery and target capability; Sustained release of drugs	[107]
	Membrane‐fusogenic liposome (MFL)‐containing hydrogel (MFL@PLGOX)	Sialyltransferase inhibitor (P‐3Fax‐Neu5Ac, PFNAc)	Melanoma	Immunotherapy	Injectable; Tumor cell membrane fusion; Natural killer cell activation	[108]
Nanoparticles loaded	Fibrin gel	aCD47@CaCO_3_	Melanoma	Immunotherapy	Sprayable; Low systemic toxicity; Promote wound healing; Promote M1‐type TAM activation	[78]
	DOX/ICG/CpG‐P‐ss‐M/CD hydrogel system	CpG‐P‐ss‐M and DOX	Melanoma	Immunotherapy; Chemotherapy; Photothermal therapy	Achieve all the stages of the LDIMP cascade; In situ tumor‐specific antigen storage; Limited systemic side effect	[79]
	QCSG/HA‐DA/ZDH cryogel	ZIF‐8/DA/HMME (ZDH) nanoparticles	Liver cancer	Sonodynamic therapy	Rapid hemostasis; Antibacterial capability; pH responsive	[80]
	GOx@MnCaP@fibrin gel	GOx@MnCaP	Glioma	Chemodynamic therapy	Sprayable; Good biocompatibility; Long‐term drug release	[81]
	D‐I@MSN/gel	DOX and ICG co‐loaded mesoporous silica nanoparticles	Liver cancer	Chemophotothermal therapy	Thermosensitive; Low systemic toxicity; Enhanced delivery and target capacity	[82]
	PTX‐NPs‐DN hydrogel	Paclitaxel (PTX) nanoparticles	Breast cancer	Chemotherapy	Local and sustained delivery of drugs; Re‐filling and supporting the tumor resection cavity	[83]
	PEG‐DMA hydrogel	PTX PLGA nanoparticles	Glioblastoma	Radiotherapy; Chemotherapy	Efficient encapsulation of poorly soluble drugs; Sustained drug release; Biodegradability and biocompatibility	[115]
	PPNPs/EPB@HA‐Gel	PTX nanoparticles	Breast cancer	Chemotherapy	Injectable; Reducing systemic toxicity; Steadily release of the encapsulated drugs; Low systemic toxicity	[116]
	CS@BPNSs@CuNPs hydrogel	Black phosphate nanosheets (BPNSs) and copper nanoparticles (CuNPs)	Glioblastoma	Chemodynamic therapy; Immunotherapy	Rapid hemostasis; Promote wound healing; Anti‐bacterial property	[117]
	Gel‐SA‐CuO	CuO nanoparticles	Liver cancer	Photothermal therapy	Biodegradability and biocompatibility; Ferroptosis induction; Drug retention and controlled release	[118]
	CO–HA Gel	Curcumin‐loaded polymeric nanoparticles	Uveal melanoma	/	Retention and sustained release of drugs; Easy administration; Prevents drug/particle migration	^[^ ^119^ ^]^
	PDNPs‐gel	Peptide (CRGDK)‐modified DOX‐based prodrug nanoparticles	Breast cancer	Chemotherapy	Injectable; In situ thermosensitive self‐gelation; Enhanced delivery and target capability; Biocompatibility and biodegradation	[120]

## 4 OTHER POSTOPERATIVE APPLICATIONS OF GEL/HYDROGEL

Apart from preventing tumor recurrence and metastasis, in situ biomaterial platforms also play unreplaceable roles in other post‐surgical applications such as hemostasis, antibacterial infection, tissue adhesion, tissue repair, and wound healing. Additionally, in situ biomaterial platforms with multifunctions have been developed, aiming at achieving better and faster recovery after surgery.

### 4.1 Hemostasis

Hemostasis is the process of platelet aggregation, degranulation, and thrombus formation, which is an important step of postoperative treatment.^[^ ^121^ ^]^ If the bleeding is not well controlled, surgical hemorrhage may lead to serious consequences such as hypovolemic shock, hypothermic coagulopathy, or even death.^[^ ^122^ ^]^ Traditional hemostatic materials such as gauzes and bandages have been used for simple hemostasis of superficial wounds. However, when involving vital tissues or organs (e.g., brain) that compressive hemostasis would cause damages to the functions, the traditional techniques are at a loss. Compared to the traditional hemostatic materials, in situ biomaterials such as hydrogels may be a better option, whose injectability and flowability enable their applications to irregular wounds and intracavity injuries, which can be adapted to complex environment and achieve rapid and effective hemostasis.^[^ ^123^ ^]^ Tavakoli et al. developed a Kappa carrageenan (κCA)‐coated starch/cellulose nanofiber hydrogel with adjustable degradation rate and blood clotting ability.^[^ ^124^ ^]^ The hydrophilic functional groups in hydrogels had the capability to absorb a considerable amount of water and maintain them inside the 3D network, resulting in the swelling of hydrogels to pad the injured cavity. Simultaneously, the swollen hydrogel provided a high moisture environment around the wound site, which promoted the cellular function of angiogenesis to contract the wound and accelerated hemostasis.^[^ ^125^ ^]^ Additionally, the κCA‐modified hydrogel membrane had excellent physiological stability with a two times reduced degradation rate compared to traditional hydrogels, which became an ideal candidate for hemostatic materials. Also, rapid hemostasis after tumor surgical resection is essential for inhibiting tumor recurrence and metastasis as bleeding at resection sites may carry residual tumor cells to circulate in the bloodstream.^[^ ^126^ ^]^ To solve this problem, Li's group developed a novel hemostatic biomaterial with a rich porous structure that can absorb the disseminated tumor cells stuck with the clotted blood to inhibit metastasis.^[^ ^127^ ^]^ There are various synthetic routines and numerous optional substances for hydrogel fabrication, which provide plenty of room to design biomaterial platforms with expected properties and functions to adapt to the in vivo environment. Hence, in situ hydrogel platforms show tremendous potential in postoperative application.

### 4.2 Antibacterial infection

Surgical site infections (SSIs) are one of the reasons that lead to poor prognosis. The most common pathogen is *Staphylococcus aureus*. Although sterilization methods and systemic antimicrobial prophylaxis have been applied before and during surgery, SSIs are still big challenges that need to be overcome after surgery.^[^ ^128^ ^]^ Malizos et al. pointed out that SSIs served as one of the main reasons for orthopedic implant failure, which may cause septic complications, and under more severe situations the implant needs to be removed, or it may cause and increased death rate otherwise.^[^ ^129^ ^]^ Therefore, they developed a fast‐resorbable hydrogel formed by hyaluronan and poly‐d,l‐lactide covalent linking. Subsequently, the hydrogel was further coated by a network loaded with various antibacterials and antibiotics. The antibacterials and antibiotics that are compatible with the hydrogel include rifampicin and ciprofloxacin. They were first mixed in a solution, then spread onto the surface of the implant before insertion. The reconstitution of hydrogel from sol‐to‐gel only took a few minutes, afterward the antibacterial coating formed on the implant surface. During the following 48–72 h, the hydrogel would undergo complete hydrolytic degradation in vivo and release the loaded antibacterials and antibiotics to prevent antibacterial infection. This application of antibacterial infection hydrogel implant coating significantly reduced the rate of postoperative infection.

When treating the drug‐resistant bacteria, such as methicillin‐resistant *S. aureus*, the traditional inherent antibacterial components alone cannot eradicate them. Hence, more effective combination therapies have been developed to combat postoperative bacterial infections. For example, Sun et al. designed a polydopamine nanoparticle‐coupled PEG hydrogel that could rapidly solidify at the wound site after spraying and induce a photothermal effect to produce ROS under near‐infrared irradiation, which could realize the local pathogen suppression and the antibacterial infection effect.^[^ ^130^ ^]^ Gonsalves et al. designed an in situ forming nanocomposite hydrogel (NCH) composed of PLGA‐carboxymethyl chitosan nanoparticles loaded with the chemotherapeutic drugs DOX and 5‐fluorouracil for skin cancer postoperative antibacterial activity and residual tumor cells elimination.^[^ ^131^ ^]^ The experimental results exhibited promising bacterial inhibition effect against *S. aureus* and *Escherichia coli* of NCH containing drug‐loaded nanoparticles group, and statistically significant cytotoxic of anticancer drugs‐loaded nanoparticles group to human epidermal carcinoma A431 and melanoma G361. In summary, the application of antibacterial drug‐loaded gel/hydrogel platforms effectively eliminates SSIs and relieves the pain of patients, which may be a promising solution to improve operative prognosis.

### 4.3 Adhesion prevention

Post‐surgical tissue adhesion is a serious complication that can lead to poor prognosis such as limited flexibility and impaired functions of tissues or organs, which is caused by the interweaving of fibrin bands between surgical sites and surrounding tissues or organs.^[^ ^132^ ^]^ Currently, utilizing barrier materials to physically separate the injury sites and their adjacent tissues is the most effective method of postoperative adhesion prevention. The ideal properties of anti‐adhesion materials should be (1) biocompatible and non‐cytotoxic; (2) tightly adhesive to tissue surface; (3) stably exist in vivo in the first several days and self‐degradable within 1 month; (4) ease of handle and control. As the in situ hydrogel platforms perfectly fit all these requirements, they have great potential to be the suitable barrier materials for tissue adhesion prevention.^[^ ^133^ ^]^ In recent years, researchers have successfully developed anti‐adhesion hydrogel platforms. For example, Mizuno et al. designed a decyl group‐modified gelatin (C10‐ApGltn) and a poly (ethylene glycol)‐based anti‐adhesion hydrogel for colorectal surgery.^[^ ^134^ ^]^ Stapleton et al. developed a supramolecular polymeric hydrogel as an effective adhesion barrier for post‐pericardial surgery application.^[^ ^135^ ^]^ Adhesion prevention is also crucial after tumor resection, especially for abdominal tumors. Zhou et al. developed a hydrogel‐based drug delivery system to prevent intraperitoneal adhesions after cervical cancer operation.^[^ ^136^ ^]^ In general, the application of in situ hydrogel platforms at surgical sites ideally prevents the adhesion to surrounding tissues, which is essential in postoperative management and has great potential for further clinical translation.

### 4.4 Tissue repair and wound healing

**FIGURE 8:**
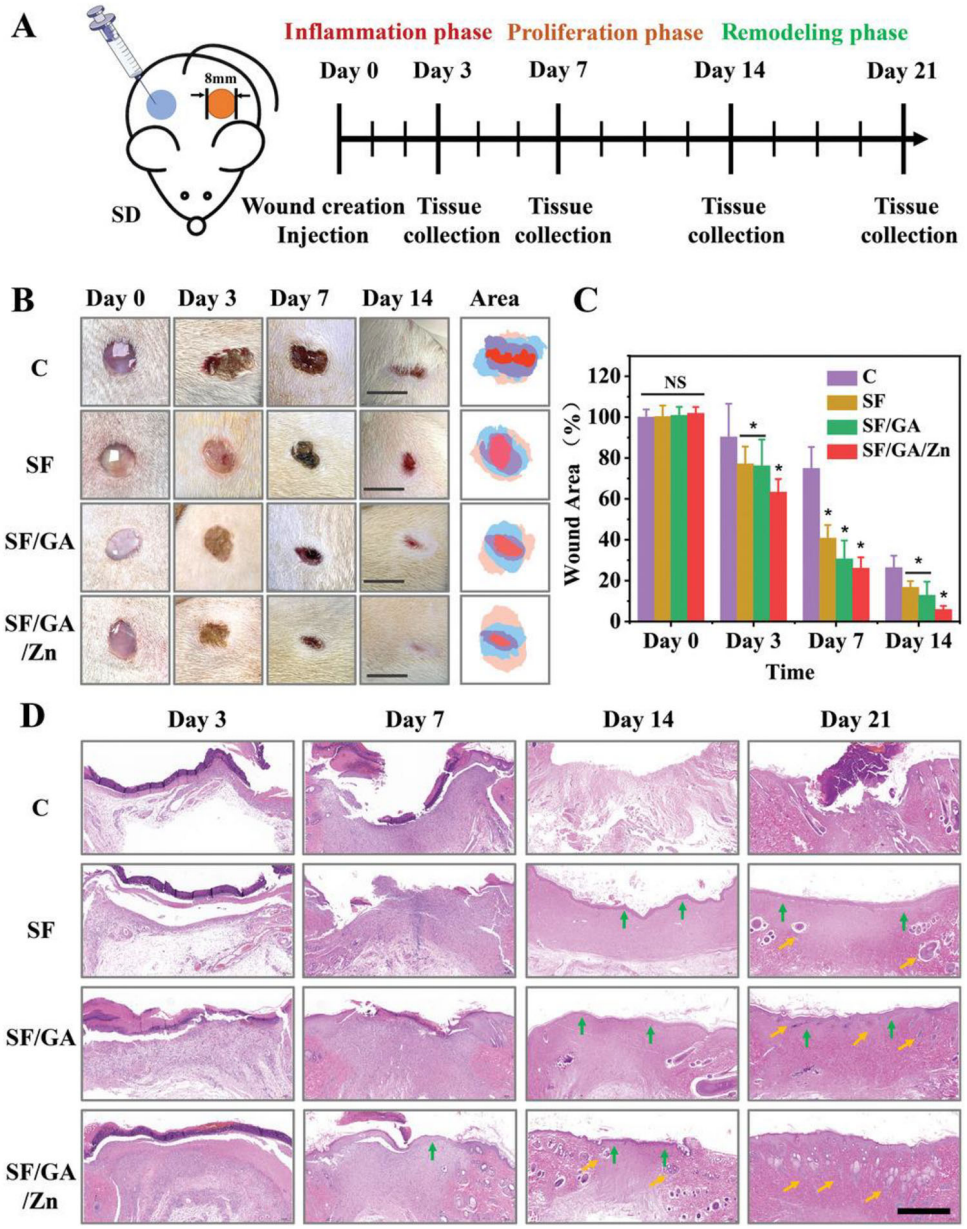
Effects of hydrogel in diabetic wound healing promotion. (A) Design of diabetic mice model to test wound healing capacity of hydrogel. (B) Representative photographs of the wound healing process in different groups. Scale bar: 10 mm. (C) The statistical analysis of the wound area. (D) H&E staining of collected wound tissues at days 3, 7, 14, and 21. The green arrows and yellow arrows indicate the re‐epithelialization area and newly formed dermal respectively. Scale bar: 500 μm. Reproduced with permission from reference.^[^ ^146^ ^]^ Copyright 2022, Wiley‐VCH GmbH.

Tissue repair and wound healing are highly coordinated physiological processes for damage reparation and function restoration, which play a significant role in postoperative recovery.^[^ ^137^ ^]^ For tissues and organs that undergo re‐epithelialization during reparation (e.g., skin, lung, and kidney), the injured wound attempt to maintain the integrity of epithelium by wound healing.^[^ ^138^ ^]^ The complete process of wound healing consists of four phases: hemostasis, inflammation, proliferation, and tissue remodeling.^[^ ^139^ ^]^ The inflammation phase occurs immediately in response to the injury, triggering the localized release of inflammatory mediators that result in the phagocytic leucocytes influx to degrade devitalized tissues. In the next proliferation stage, the wound starts to rebuild to the original state by synthesizing collagen and ECM for granulation tissue construction, and angiogenesis also occurs for nutrition supply. After the wound defect is filled completely and the newborn epithelial cells fully resurface the wound, it comes to the final remodeling stage, during which the wound regains its tensile strength and recovers to the original state of blood supply.^[^ ^140^ ^]^ To facilitate wound healing, one effective method is to apply in situ biomaterial platforms loaded with relative factors which can accelerate some of the processes involved in the four stages. For example, Notodihardjo et al. utilized platelet‐rich plasma releasate embedded in gelatin sheets to promote wound recovery in the proliferation stage.^[^ ^141^ ^]^ The platelet‐rich plasma is plasma containing concentrated platelets, which would release various growth factors with activation, including platelet‐derived growth factor, VEGF, and transforming growth factor beta. These released factors from in situ biomaterial platform contributed to accelerating wound healing. Additionally, the hollow scaffold structure of hydrogel could improve cell proliferation and tissue regeneration. Utilizing this advantage, Liu et al. synthesized hydrogel/PCL core/shell fiber scaffolds to promote wound healing after breast cancer resection and kill the residual tumor cells to inhibit recurrence.^[^ ^142^ ^]^ Other than loading growth factors to facilitate wound healing, in situ biomaterial platforms can also be designed for patients with specific needs. For instance, wound healing for patients suffering from diabetes mellitus is hard because of hyperglycemia. The hyperglycemic environment can disrupt skin barrier and impair immune function, therefore increasing bacterial diversity and promoting biofilm formation, which hinders the process of wound healing.^[^ ^143^ ^]^ The impaired function of wound healing not only leads to complications such as ulceration in the lower limb,^[^ ^144^ ^]^ it could also be a great concern when diabetes patients need to undergo surgical resection. Under this circumstance, personalized design of in situ biomaterial platforms loaded with specific medical or non‐medical could be a promising solution.^[^ ^145^ ^]^ Utilizing the strategy that modulates the polarization of macrophage from M1 to M2 in diabetic wounds, Qian et al. designed a glycyrrhizic acid (GA)‐based hybrid hydrogel composed of Zn^2+^ and methacrylate silk fibroin (SFMA).^[^ ^146^ ^]^ The Zn^2+^ assisted to maintain immune functions while the combination of SFMA facilitated macrophage polarization. Taken together, this hybrid hydrogel significantly accelerated diabetic wound healing and induced the generation of dermis in mice model compared to the control group (Figure 8), indicating the essential role of hydrogel in wound healing promotion.

In the process of tissue repair, scar formation is a normal result of wound healing because of fibrogenesis, the generation of new collagen fibers, which normally will not cause side effects except the influence of beauty. However, for some tissues, the scar formation can severely limit the repair of their functions or even lead to treatment failure.^[^ ^147^ ^]^ In recent years, researchers have utilized in situ biomaterial platforms to limit postoperative scar formation. Choi et al. designed a hepatocyte growth factor (HGF)‐loaded hyaluronic acid and alginate (HA/ALG) hydrogels to prevent and repair the VF scar formation.^[^ ^148^ ^]^ VFs are the connective tissues with layered structures that are made up of a complex ECM, which play an important role in determining voice quality. Injury of VF such as surgical wounds would cause the formation of VF scar, which may affect the capability of VF to vibrate and lead to dysphonia. Hence, uncontrolled VF scar formation is an unneglectable concern especially for the professions depending on voice, such as singers and hosts. HGF has been proved to reduce collagen production from VF fibroblasts by stimulating HA synthesis. With the delivery by in situ HA/ALG hydrogels, the sustained release of HGF on the administrated site significantly meliorate the formation of VF scar. Additionally, Chun et al. introduced another biomaterial platform application to prevent post glaucoma filtration surgery (GFS) scarring caused by excessive deposition of collagen.^[^ ^149^ ^]^ The post‐GFS fibrosis commonly occurs, largely limiting the therapeutic effect or even leading to treatment failure. Small interference RNA (siRNA) therapy has been proven to efficiently prevent and treat fibrotic disorders by silencing pro‐fibrotic genes including collagen I and TGF‐β_2_. Here they designed an siRNA that targeted on a fibrosis‐related gene which is secreted protein, acidic and rich in cysteine (SPARC). The siRNA for SPARC (siSPARC) were encapsulated inside the positively charged tuned gelatin hydrogel that prevented siRNA from degradation, which showed significant reduction in subconjunctival scarring without cytotoxicity after GFS in rabbit model. Basically, hydrogels that are capable of inhibiting collagen production can minimize scar formation, which might be the most suitable postoperative biomaterials to treat patients with voice‐dependent occupations or some vital organs.

Bone healing is another type of tissue repair which is unique in connective tissue. After accidental injury or surgical drilling, the bone will go through a complex regeneration process with the initiation of inflammatory response, followed by a series of physiological processes including angiogenesis, osteogenic differentiation, and biomineralization.^[^ ^150^, ^151^ ^]^ In the first state of inflammatory response, macrophages polarize into M1 phenotype for clearance and phagocytosis. In the following 3 days, macrophages will polarize from M1 to M2 phenotype to induce bone regeneration.^[^ ^152^ ^]^ However, severe damage to bone may recruit a large amount of cytotoxic T cells, which prolongs the inflammation response and delays bone healing.^[^ ^153^, ^154^ ^]^ Utilizing this mechanism, Fan and his group developed an ECM hydrogel derived from periosteum for the optimization of bone healing process.^[^ ^155^ ^]^ Compared to traditional decellularized scaffolds, which are made up of dense collagen fiber network with low cell permeability and lack of proinflammatory response regulation,^[^ ^156^ ^]^ the periosteal ECM hydrogel precisely modulated M1‐to‐M2 macrophage polarization and exhibited spontaneous biomineralization property that contributed to successful and rapid bone healing.^[^ ^157^ ^]^ In summary, hydrogel platforms are promising biomaterials that can not only accelerate the repair of different types of tissues and promote wound healing but also minimize scar formation, which can be flexibly designed to meet the requirements of different patients.

### 4.5 Multifunctional in situ hydrogel platforms

To meet different demands of postoperative treatment, in situ biomaterial platforms with various functions have been developed. Furthermore, some researchers have successfully combined two or more functions into one in situ biomaterial platform, achieving comprehensive postoperative treatment and recovery by realizing synergic effect within one multifunctional in situ biomaterial platform. Zhao et al. developed an injectable hydrogel system with multifunctional properties, which significantly promoted the postoperative wound healing process in vivo and helped fast recovery of tissue morphology and function.^[^ ^158^ ^]^ The hydrogel is composed of quaternized chitosan‐g‐polyaniline and benzaldehyde group functionalized poly(ethylene glycol)‐*co*‐poly(glycerol sebacate), serving as the electroactive dressing for cutaneous wound healing, which presented rapid self‐healing ability, efficient hemostasis capacity, and good adhesiveness to tissues. Additionally, this injectable hydrogel exhibited excellent antibacterial infection capability, biocompatibility, and free radical scavenging ability because of the introduction of the polyaniline onto quaternized chitosan. Last but not least, this self‐assembled hydrogel showed rapid gelation capability with high mechanical strength, endowing it with soft and flexible properties. Taken together, the design of multifunctional hydrogel systems fully realized the advantages of in situ biomaterial platforms and achieved an ideal outcome for postoperative treatment, which deserved more attention and excavation in the future.

In summary, in situ hydrogel biomaterial platforms have the capability to meet several needs and assist each recovery process in postoperative management. Some representative examples are summarized in Table 2. Because of their superb biocompatibility and injectable capability, in situ formed hydrogel can fit the shape of wound and the released factors from hydrogels can induce blood clotting for rapid hemostasis. When loading antibiotics in the platforms, the application of hydrogels can eliminate bacterial infection at surgical sites. In addition, the hydrogels can separate surgical sites from surrounding tissues, which prevents postoperative tissue adhesion that may lead to poor prognosis. Furthermore, hydrogel platforms can promote tissue repair and wound healing by provide a high moisture environment and growth factors that are beneficial for recovery. Last but not least, the in situ hydrogel platforms can be flexibly designed with multifunction to meet personalized requirements, which exhibit huge advantages compared to traditional biomaterials and provide better postoperative recovery and experience for patients.

**TABLE 2:** Summary of representative hydrogel biomaterial platforms in other postoperative application including their names, loading components, applications, and advantages

Name	Loading components	Application	Advantages	Reference
Kappa carrageenan (κCA)‐coated starch/cellulose nanofiber	/	Hemostasis	Enhanced mechanical strength; Reduced degradation rate and swelling ability	[124]
Defensive antibacterial coating, DAC^®^	Antibiotics	Antibacterial infection of implants	Fast resorbable; Rapid degradation to release antibiotics; Without interfering with bone growth	[129]
PEG‐PDA hydrogel	Polydopamine nanoparticle (PDA NP)	Antibacterial infection	Sprayable; Rapid in situ gel forming; Photothermal responsive	[130]
Poly(ethylene glycol‐maleate‐citrate) (PEGMC)‐based nanocomposite hydrogel	Doxorubicin (DOX) and 5‐fluorouracil (5‐FU)‐loaded poly lactic‐*co*‐glycolic acid (PLGA)‐carboxymethyl chitosan (CMC) nanoparticles	Antibacterial (*Staphylococcus aureus*) infection	Injectable; pH responsive; Promote wound healing; Prevent skin cancer postoperative recurrence	[131]
Decyl group‐modified gelatin (C10‐ApGltn) based hydrogel (C10‐gel)	/	Tissue adhesion prevention	Prevent anastomotic leakage; In vivo degradation	[134]
Polymer–nanoparticle (PNP) hydrogels	Poly(ethylene glycol)‐*b*‐poly(lactic acid) (PEG–PLA) nanoparticles	Pericardial adhesion prevention	Tunable viscoelastic mechanical properties; Excellent biocompatibility; Rapid self‐healing	[135]
Collagen‐APG6K‐cysteine (Col‐APG‐Cys) @HHD hydrogel	HAS‐18His protein and docetaxel (HHD) nanoparticles	Abdominal adhesion prevention	Chemo drug loaded; Prevent tumor recurrence	[136]
Gelatin hydrogel	Platelet‐rich plasma releasate (PRPr)	Promote wound healing	Promote neoepithelialization and wound contraction; Promote angiogenesis; Cheap	[141]
Photo‐cross‐linked methacrylate silk fibroin/glycyrrhizic acid/Zn2+ (SF/GA/Zn) hybrid hydrogel	/	Promote diabetic wound repair	Excellent injectability and mechanical strength; Immunoregulatory activity; Ease of preparation	[146]
HGF–hyaluronic acid and alginate (HA/ALG) composite hydrogels (HGF–HA/ALG)	Hepatocyte growth factor (HGF)	Facilitate vocal fold (VF) wound healing	Ameliorate VF scarring	[148]
Gelatin‐tyramine (Gtn‐Tyr) hydrogel	Small interference RNA (siRNA) target secreted protein, acidic and rich in cysteine (siSPARC)	Anti‐scarring therapy in post glaucoma filtration surgery	Positive‐charge tuned; Protect siRNA from degradation; Deliver siRNA effectively; Simple fabrication and highly scalable	[149]
Periosteal extracellular matrix (PEM) hydrogel	/	Promote bone repair	Injectable; Enhance angiogenesis and osteogenesis; High cell permeability; Safe; Non‐toxic; Multi‐effective	[155]
Quaternized chitosan‐g‐polyaniline (QCSP)/poly(ethylene glycol)‐*co*‐poly(glycerol sebacate) (PEGS)‐4‐formylbenzoic acid (FA) (QCSP/PEGS‐FA) hydrogel	/	Promote cutaneous wound healing	Antibacterial; Anti‐oxidant; Electroactive; Injectable; Promote hemostasis; Self‐healing capacity; Free radical scavenging capacity	[158]

## 5 CONCLUSION AND PROSPECTS

In this review, we give an overview of the properties, advantages, and synthesis methods of the in situ gel/hydrogel‐based platforms and their applications in cancer postoperative treatment and recovery. For a start, we briefly introduce the current development status of the cancer therapeutic effect after tumor surgical resection, which usually faces with the risk of tumor local recurrence and distant metastasis that result in cancer treatment failure. Additionally, complications may appear at the operative site if they cannot be treated in a good manner. Then, we point out the potential of the in situ biomaterial platforms which are regarded as promising adjuvant anticancer strategy, especially the gel/hydrogel‐based platform. The properties including good biocompatibility, adaptive shapes, controlled drug release, and rapid preparation, and their synthesis procedures are introduced in detail. Meanwhile, the advantages such as limited systemic side effects and prolonged drug release significantly help increase the postoperative therapeutic efficiency, compared to traditional therapies. Then, the designable drug loading category in gel and hydrogel, based on the classification of drugs, including anticancer drugs, immunologic agents, cell components, and nanoparticles are discussed. When handling with more strict conditions, such as drug‐resistant tumors and immunosuppressive TME, combination therapies, such as chemodynamic therapy, immunotherapy, photodynamic therapy, and thermodynamic therapy, can be realized in the platform, aiming at achieving synergetic anti‐tumor effect. Finally, the applications of the in situ gel/hydrogel platform for postoperative recovery were introduced, including hemostasis, antibacterial infection, adhesion prevention, tissue repair, and wound healing, which provide promising assistance and guarantee rapid and comprehensive postoperative rehabilitation of cancer patients.

Despite the merits of in situ gel/hydrogel platforms, there are some challenges to be explored for future development. First, although some gel/hydrogel composed of natural ingredients have relatively higher biocompatibility, they still need to include other synthetic molecules for cross‐linking, whose metabolites may be toxic to healthy tissues and cause local side effects. In addition, the chemical modification of molecules during the synthesis routine may also cause similar concerns when they are applied to physiological environment. Qian et al. proposed the idea of developing a pure molecular hydrogel by raltitrexed self‐assembly, which may be one solution to resolve this challenge.^[^ ^159^ ^]^ Second, high water content may increase the permeability of hydrogels, leading to a burst release of drugs. The high concentration of drugs can cause acute toxicity to local and surrounding tissues, and decrease the therapeutic efficacy of hydrogel platforms at the same time. Researchers should balance water content and control the release rate of drugs to realize long‐term treatment and reduce local toxicity. Third, the design of in situ gel/hydrogel platforms is complicated, and the synthesis cost is expensive. Future research should focus on developing rapid and easy preparation methods of gel/hydrogel platforms with low cost, which is an important prerequisite for future promotion in mass production and clinical translation.

## CONFLICT OF INTEREST STATEMENT

The authors declare no conflict of interest.
